# Bibliometric Analysis on Equine-Assisted Interventions

**DOI:** 10.3390/ani14121776

**Published:** 2024-06-13

**Authors:** María Amado-Fuentes, Angel Denche-Zamorano, Sabina Barrios-Fernandez, Margarita Gozalo

**Affiliations:** 1 Psychology and Anthropology Department, University of Extremadura, 10003 Caceres, Spainmgozalo@unex.es (M.G.); 2 Promoting a Healthy Society Research Group (PHeSO), Faculty of Sport Sciences, University of Extremadura, 10003 Caceres, Spain; 3 Occupation, Participation, Sustainability and Quality of Life (Ability Research Group), Nursing and Occupational Therapy College, University of Extremadura, 10003 Caceres, Spain

**Keywords:** equine-assisted services, horses, education, therapy, scientometrics

## Abstract

**Simple Summary:**

The present work presents a snapshot of the state of the art of Equine-Assisted Intervention (EAI) research, including published articles and the most cited research as well as the most used keywords, and prolific authors and co-authors and their countries. To our knowledge, no other bibliometric studies have been performed, so it can be a tool to support future research.

**Abstract:**

Equine Assisted Interventions (EAIs) integrate the active participation of horses in therapeutic or educational interventions. A bibliometric analysis was carried out on this topic, using traditional bibliometric laws and recommendations. For this purpose, a search on the Web of Science (WoS) Core Collection database was carried out, obtaining 333 documents. Annual publications followed an exponentially increasing trend (R^2^ = 86%), pointing out that this topic is a growing interest among researchers, publishers, and journals. The USA was the most productive country worldwide and Jeong-yi Kwon and Ji Lee were the prolific co-authors. The WoS category with the highest number of papers was Rehabilitation (84 papers). The *Journal of Alternative and Complementary Medicine*, and *Pediatric Physical Therapy* were the journals with the highest number of publications. The most cited paper was “State of the Evidence Traffic Lights 2019: Systematic Review of Interventions for Preventing and Treating Children with Cerebral Palsy”. The most used author keywords were rehabilitation, balance, and those related to specific populations such as Cerebral Palsy and Autism Spectrum Disorder. These results suggest that EAIs is a topic of increasing interest for researchers, editors, and professionals.

## 1. Introduction

According to the non-profit organization Animal Assisted Intervention International, Animal-Assisted Interventions (AAIs) is an interdisciplinary term which describes different types of activities with animals in various fields of action (education, health, and social), with different objectives, populations (ages and disabilities) and settings [1]. The history of AAIs spans diverse cultures and temporal periods. Although there is no exact date for their foundation, there is historical evidence that ancient civilizations, such as the Greeks and Romans, recognized the positive effects of interaction with animals on human health. Animals such as dogs and horses were used for therapeutic purposes, although in a less structured way than today [2]. For example, in the American Civil War (1861–1865), horses and dogs were used for the rehabilitation of wounded soldiers and those with post-traumatic stress disorder [3]. During the 20th century, AAIs began to develop in a more formal and structured manner. Both world wars expanded equine therapies as an alternative therapeutic approach. At the end of World War I in Great Britain, Olive Sands offered her horses to The Oswestry Orthopedic Hospital (1901) to benefit patients, with positive results. Moreover, the first equine therapy group was established at the Oxford University Hospital for wounded people in this war [3].

Today, AAIs have gained international recognition and are applied in a variety of settings, including mental health, special education, occupational therapy, and rehabilitation. Depending on the goals, EAI is led by health or educational professionals [4,5]. With the growth of animal welfare awareness, there has been an increasing emphasis on ensuring that interventions are conducted ethically and respectfully toward the animals involved [6].

Equine Assisted Interventions (EAIs) are specialized programs that integrate the presence and active participation of horses in therapeutic or educational interventions. These programs are carefully designed to address a wide range of needs and goals in areas such as physical health, rehabilitation, emotional development, and personal growth [7,8]. These services have demonstrated benefits at a physical level with improved trunk control, balance, strength, and gross motor function [9,10,11]; mental health and cognitive aspects [12,13,14,15,16]; and learning-positive effects in behavior and learning and daily living skills [17,18,19]. Due to the diversity of terminology used, there is a large number of terms that have been used as synonyms. For this reason, Wood and collaborators in coordination with The Professional Association of Therapeutic Horsemanship International (PATH Intl.) reached a consensus on the types of activities with horses, establishing a generic term, Equine-Assisted Services (EASs) [20]. Within EASs, there are three groups depending on the objective and the specialist performing the activity: (1) therapy, led by health professionals who integrate horses into therapeutic programs or interventions to enhance health and well-being; (2) learning and education, led by educational and social professionals, incorporating horses into experiential learning activities with horses; and (3) riding, guided by equestrian sports technicians, with practices oriented to adapted traditional equestrian disciplines such as dressage, vaulting, and driving. However, this terminology was not supported by organizations such as the American Hippotherapy Association (AHA). Due to the lack of articles with the word “services”, the term “interventions” was used throughout this paper.

Bibliometric studies are investigations that focus on the quantitative analysis of scientific and academic production. These studies use bibliographic data, such as the number of publications, citations, scientific journals, and other indicators, to evaluate and measure the activity and impact of research in a specific area [21,22], which is helpful for researchers and publishers, and therefore for the topic of the study itself [23], EAIs or EASs, and its terminology, scope, or domain, the set of human–animal interactions or interventions has not been able to bring the main authors or organizations into an agreement. This is why this has been reflected in the introduction, hoping it will be beneficial for researchers and technicians to have a starting point in new research or the search for papers. To our knowledge, no bibliometric studies on EAIs or related topics have been carried out, but systematic reviews in specific populations, such as in Autism Spectrum Disorders (ASDs) [24] or Cerebral Palsy [25], exist. Therefore, this study aims to explore the current scientific situation of EAI publications, including the annual publication trend, prolific co-authors and relationships between them, the most cited documents, the journals with the highest number of publications, and the most used author keywords.

## 2. Materials and Methods

### 2.1. Design and Data Source

A descriptive bibliometric analysis was conducted to map the scientific research published on EAIs in journals indexed in the Web of Science (WoS), the most used source for this kind of study [26]. The WoS is a platform formed by a wide collection of bibliographic databases, citations, and references of scientific publications of any discipline of knowledge that, in addition to providing bibliographic information, allows to evaluate, and analyze the performance and scientific quality of research. Data were obtained by using the WoS Main Collection (Clarivate Analytics, Philadelphia, PA, USA), limiting the search to the Science Citation Index Expanded (SCI-EXPANDED), Social Sciences Citation Index (SSCI), and Emerging Sources Citation Index (ESCI) editions. The search was performed on 3 November 2023 using the search vector: ((ti=(“hippotherapy”) OR ti=(“equine assisted therap*”) OR ti=(“equine assisted service*”) OR ti=(“equine assisted activit*”) OR ti=(“equine assisted intervention*”) OR ti=(“therapeutic horsebackriding”) OR ti=(“equine assisted therapeutic activit*”) OR ab=(“hippotherapy”) OR ab=(“equine assisted therap*”) OR ab=(“equine assisted service*”) OR ab=(“equine assisted activit*”) OR ab=(“equine assisted intervention*”) OR ab=(“therapeutic horsebackriding”) OR ab=(“equine assisted therapeutic activit*”) OR ak=(“hippotherapy”) OR ak=(“equine assisted therap*”) OR ak=(“equine assisted service*”) OR ak=(“equine assisted activit*”) OR ak=(“equine assisted intervention*”) OR ak=(“therapeutic horsebackriding”) OR ak=(“equine assisted therapeutic activit*”))), without type of document or time constraints. All the documents indexed in the WoS up to the search date were included (3 November 2023). This search was designed to be restrictive, due to the terminological problems associated with EAIs. Therefore, the search string was constructed as described above, without including commands such as NEAR.

### 2.2. Data Analysis

The following exclusion criteria were applied to the retrieved papers: (1) manuscripts on animal therapies in general; (2) studies related to mechanical horses; (3) volunteers’ or families’ perceptions; (4) articles related to therapy horse training; (5) investigations where the horse is receiving veterinary therapy or treatment; (6) studies on the benefits in riding and not in therapy; and (7) on professionals’ knowledge of the therapies themselves. Two independent authors applied these criteria and a third resolved disagreements in the case of discrepancies.

Traditional bibliometric laws were followed to perform the analysis of the set of documents. Their trend and the possibility of being in an exponential growth phase were analyzed with the determination coefficient (R^2^) using de Solla Price’s law of the exponential growth of science [27,28]. Bradford’s law of science concentration was applied to detect journals with the highest number of publications [29,30,31]. Lotka’s law was applied to identify prolific co-authors [32]. The h-index was used to identify the most relevant articles in the subject area, taking h articles with h or more citations as the most relevant [33,34]. The most used authors’ keywords were analyzed by applying Zipf’s law [35]. The obtained data were downloaded in .xslx and plain text to be analyzed using Microsoft^®^ Excel^®^ for Microsoft 365 MSO version 2206 and the VoSViewer software version 1.6.18.

## 3. Results

The search yielded a total of 442 papers (340 research papers, 64 systematic reviews, and 38 others) (Supplementary Material S1). After applying the inclusion–exclusion criteria, a total of 333 manuscripts remained. Regarding the reasons for the exclusion of the 103 papers, 16 were because they were manuscripts on animal therapies in general; 22 were covering mechanical horse articles; 15 were related to volunteers’ or families’ perceptions; 21 articles related to therapy horse training; 19 were investigations where the horse is receiving veterinary therapy or treatment; 13 studies on the benefits in riding and not in therapy; and 3 because they were related to professionals’ knowledge of the therapies themselves.

**Figure 1:**
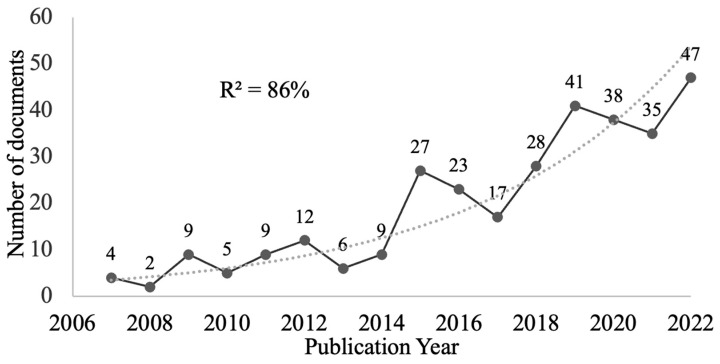
Annual publication trend.

The first document found was from 1978, although annual publications were not continuous until 2001, except in 2006. From 1978 to 1998, eight documents were published. During the following period, from 2001 to 2005, seven papers were published. There were uninterrupted publications from 2007 to 2022 (312 papers), experiencing growth that conformed 86% (R^2^) to an exponential growth rate (Figure 1). The year 2023 was excluded as it was not finished at the time of this study.

The WoS classifies all of the journals it indexes into approximately 250 groups called subject categories, and they can be classified into one or more categories. Documents were related to 66 WoS categories. The category with the highest number of documents was Rehabilitation (84 documents), doubling the number of documents compared to the second most published category, Pediatrics (41 documents). Table 1 shows the WoS categories where the greatest number of documents were concentrated.

**Figure 2:**
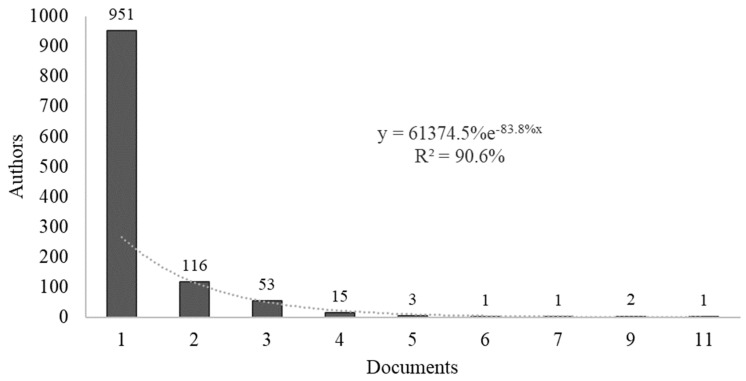
Co-authors with the most publications.

A total of 1143 co-authors produced the 333 papers analyzed. Applying Lotka’s law, it was estimated that prolific authors should be the 38 co-authors with the most publications (square root of 1143 co-authors). In the discrete count of co-authors, 76 co-authors with three or more papers and 23 co-authors with four or more papers were found, the latter being considered prolific co-authors (Figure 2).

**Figure 3:**
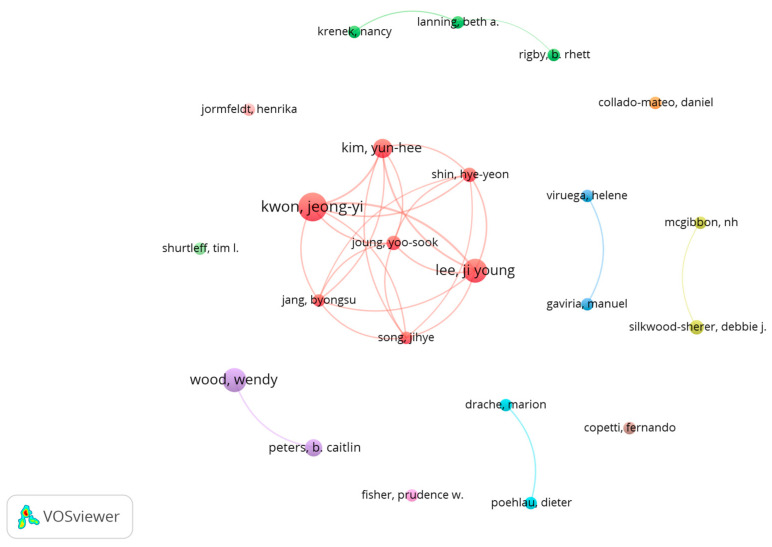
Prolific co-authors and relationships between them. Each color denotes a co-authorship cluster.

Figure 3 shows the 23 prolific co-authors and the co-authorship relationships between them. A production group consisting of seven (red cluster) of the 23 prolific co-authors was found. Other small clusters formed by two or three authors and co-authors with no connections between the rest of the prolific co-authors were also found (Figure 3).

Jeong Kwon (11 papers and 222 citations), Ji Lee (9 papers and 218 citations), and Wendy Wood, (9 papers and 113 citations), were the top three authors in terms of the number of publications, with the first two belonging to the same publication cluster. Table 2 shows the 23 prolific co-authors, production clusters formed, and number of papers and citations.

**Figure 4:**
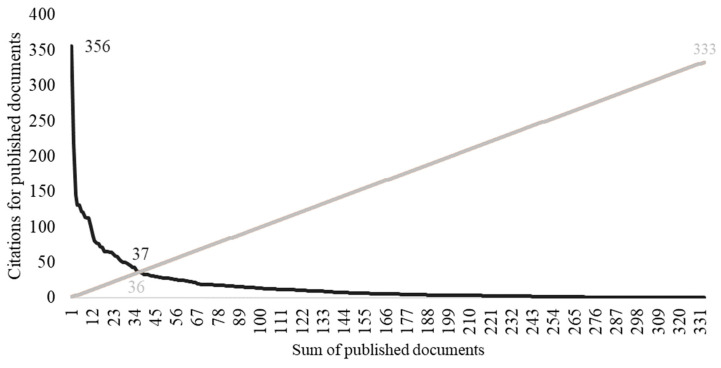
H-index estimations (cut-off points: 36 documents with 37 or more citations), where the light line is the number of documents and the black line is the number of citations.

Applying the Hirsch index (h index) to the 333 documents, 36 documents with 37 or more citations are highlighted, with these considered as the most cited and most recognized documents on the topic (Figure 4).

Table 3 shows the 36 most cited papers, their main author, the journal in which they were published, the WoS number of citations, the year of publication, the WoS index in which they are indexed, and the thematic categories to which they are related.

**Figure 5:**
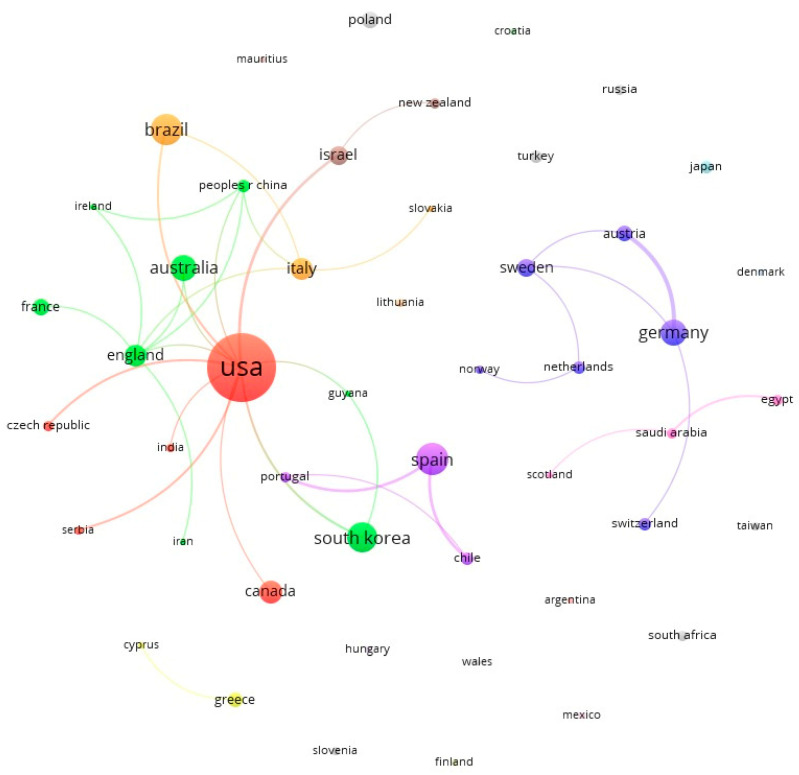
Countries/regions co-authorship graph. Colors represents the geographical co-authorships.

Figure 5 shows the geographical co-authorships, that is, the global collaboration for the production of the 333 documents. There were 49 countries/regions in co-authorship, with the USA (112 documents), Spain (25 documents), Brazil (24 documents), South Korea (22 documents), Australia (17 documents), and Germany (16 documents) being highlighted. The most productive cluster, the red one, consists of the USA together with nine other countries.

The 333 documents were published in 203 journals, but the scientific output was mainly concentrated in 15. The Bradford’s zones are shown in Table 4.

Table 5 shows the 15 journals that formed the core production, with *The Journal of Alternative and Complementary Medicine*, from the publisher Mary Ann Liebert, as the journal with the highest number of published papers (16 documents).

**Figure 6:**
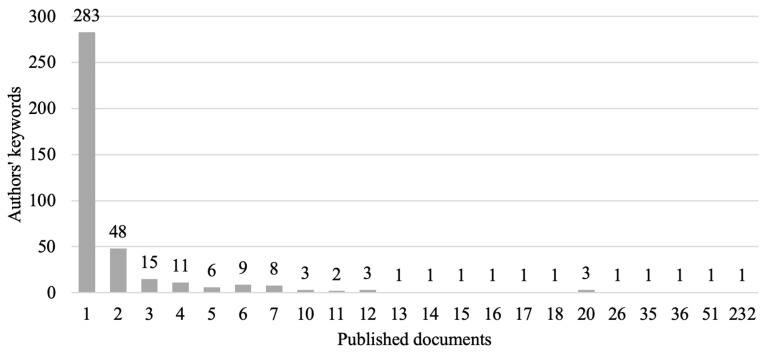
Relationship between the number of keywords that appear in each document (*x*-axis) and the frequency with which each one appears (*y*-axis).

A total of 652 author keywords were found. Once these were standardized and the duplicates removed, they were reduced to 402. After applying Zipf’s law to the 402 keywords, the most relevant keywords were estimated to be the 20 most used keywords (square root of 402). There were 19 keywords with 11 or more uses in the documents analyzed, and 22 keywords with 10 or more uses. The latter were considered to be the most relevant author keywords (Figure 6). 

Figure 6 shows the distribution of keywords by document. The *y*-axis indicates the number of keywords that exist according to the number of documents in which they appear, and the *x*-axis indicates the frequency with which they appear in the published documents.

A total of 283 keywords appear in a single document, 48 keywords appear in two documents, and so on up to the keyword that appears in the largest number of documents. This figure makes it possible to check that Zipf’s law is correctly applied. In addition, as there are a large number of keywords that are rarely used, it is useful to study those that are used frequently.

**Figure 7:**
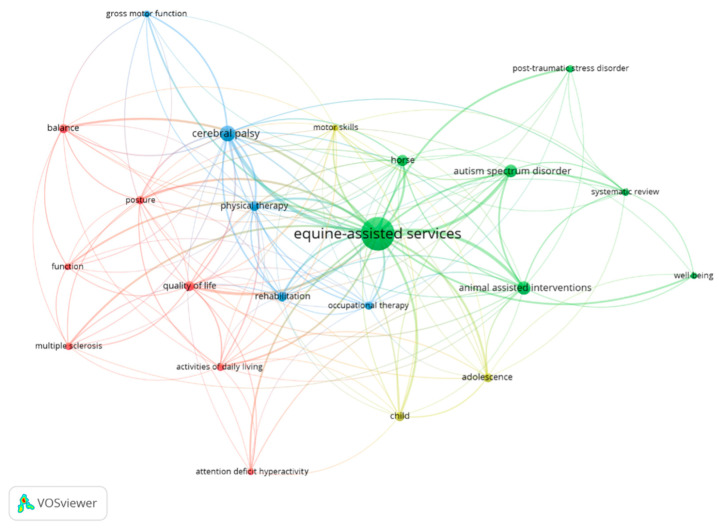
Most relevant author keyword co-occurrence graph. Colors shows the most relevant author keywords in four thematic clusters.

Figure 7 shows the most relevant author keyword co-occurrence graph. These 22 keywords generated four thematic clusters: the green one, related to well-being in neurodevelopmental and psychiatric disorders; the yellow one, more related to motor development in the early stages of life; the red one, to motor control and functionality in daily life; and the blue one, to neurological aspects and pathology.

## 4. Discussion

The relevance of this study lies in the fact that no other bibliometric studies focused on the contributions of EAIs exist. For this reason, it can be useful for researchers, publishers, and professionals interested in this type of intervention. Different indicators highlight a growing interest in this topic, with a growth in rate of publications on the subject; from 2007 to 2022, the annual publications did not stop, experiencing a growth that was adjusted by 86% (R^2^) to an exponential growth ratio.

Evidence syntheses included mapping, narratives and systematic reviews, and meta-analyses, revealing 15 reviews among the 37 most cited papers, out of which only some, “Therapeutic Effects of Horseback Riding Interventions A Systematic Review and Meta-analysis” [36] and “What is hippotherapy? The indications and effectiveness of hippotherapy” [37] are aimed at assessing the overall therapeutic effects of these interventions. Most of them focus on their effectiveness on concrete variables in specific populations. People with Cerebral Palsy receive attention in this area, with works such as Zadnikar and Kastrin (2011) [38] that focus on postural control, Whalen and Case-Smith’s work on gross motor skills (2012) [39], or the most cited article (356 citations) “State of the Evidence Traffic Lights 2019: Systematic Review of Interventions for Preventing and Treating Children with Cerebral Palsy” (Novak et al. 2020) [40], which summarizes the interventions with the best available evidence to guide researchers and practitioners. In the group of people with ASD, the title “The Effect of Therapeutic Horseback Riding on Social Functioning in Children with Autism” [41] was found with 218 citations, evaluating social function for 12 weeks with positive results. Works on people with multiple sclerosis are also among the most cited, although they receive less attention in these reviews, with less than half the number of citations of the other groups, 77 citations, in the work “Does hippotherapy improve balance in persons with multiple sclerosis: a systematic review” [42]. Among the reviews that cover more broadly the effectiveness of EAIs with different populations are works such as the one by Johnson [43] “The Benefits of Physical Activity for Youth With Developmental Disabilities: A Systematic Review”, with 130 cites. In this study, therapy with horses is included as an effective intervention among other alternatives for developing physical activity to promote health. Also noteworthy in terms of citations is the paper “Effectiveness of a Standardized Equine-Assisted Therapy Program for Children with Spectrum Disorder” (with 88 citations) by Borg [44].

The discrete count of co-authors (square root of 1143 co-authors) revealed 76 co-authors with three or more papers and 23 co-authors with four or more papers, the latter being considered prolific co-authors. The most prolific author was Jeong-Yi Kwon, Chair Professor at Sungkyunkwan University, School of Medicine (Korea) accompanied by a group of co-authors from Hankuk University of Foreign Studies (Seoul), who accounted for a large part of the productivity and made up the most cited cluster in the field (red cluster). They are followed by the group led by Wendy Wood of the University of Southern California and Caitlin Peters of Colorado State University (Occupational Therapy) (purple cluster). The rest of the authors have a much lower productivity and some of them (e.g., Collado-Mateo) cannot be associated with other prolific authors in the field.

Out of the 36 papers that stood out most concerning the h-index (cut-off: 36 documents with 37 or more citations), the paper published in 2020 by Novak et al. (2020) [40], entitled “State of the Evidence Traffic Lights 2019: Systematic Review of Interventions for Preventing and Treating Children with Cerebral Palsy”, published in the journal *Current Neurology and Neuroscience Reports* within the WoS category of Clinical Neurology; Neurosciences, with 356 citations, was highlighted. This is a systematic review of the best available evidence (2012–2019) using GRADE and the Evidence Alert Traffic Light System to add new findings to those previously made available in 2013.

The journals with the most publications in this field are *The Journal of Alternative and Complementary Medicine* by Mary Ann Liebert (United States), within the JCR category of Integrative and Complementary Medicine, ranked Q3. The second was *Pediatric Physical Therapy*, published by Lippincott Williams & Wilkins (United States) and located in Pediatrics (Q3); and Rehabilitation (Q3). In the third position was *Physiotherapy Theory and Practice* from the Taylor & Francis Group (United Kingdom) in the Rehabilitation category (Q2); and in fourth position, *The American Journal of Occupational Therapy* published by the American Association of Occupational Therapy (Rehabilitation, Q1). The journal with the highest number of citations is *Developmental Medicine and Child Neurology*, ranked Q1, with 515 citations, despite having only sevens manuscripts related to the topic. The *Journal of Autism and Developmental Disorders*, with the same number of articles, was in second position with 471 citations.

In total, 49 countries/regions were revealed in co-authorship, with the USA (112 documents) leading a wide network with other countries of great geographic dispersion. Brazil is in third place with 24 productions, followed by South Korea with 22, and other countries such as Canada and Israel. Spain’s connections (in second place with 25 documents) are more focused on other countries that are geographically close (Portugal) or for reasons of language and culture (Chile).

Among the WoS categories, the topic studied was related to 66 categories. The category to which the largest number of documents were related was Rehabilitation (84 documents), doubling the number of documents in the second most published category, Pediatrics (41 documents), followed by Clinical Neurology (36 documents), Integrative Complementary Medicine (28 documents), and Psychiatry (24 documents).

Among the authors’ keywords, the first subjects that emerge together with psychomotricity are those related to rehabilitation and psychomotor development, while the current trend focuses on physical activity and early childhood education. This is reinforced by the results obtained in the most cited papers, with half of them carried out in this population. However, this contrasts with the fact that within the journals publishing the most papers on the subject (within Bradford’s core), only one was specifically focused on the child population. Therefore, this subject could be of interest to child-specific journals, but focused on psychomotor aspects related to specific disorders or diseases. However, the current trend includes keywords from studies focused on children, on topics related to play, activity, and/or physical education and motor development.

Concerning the most frequently used author keywords, it can be seen that a large number of low-frequency keywords were found; 283 were used only once and 48 were used only twice. This dispersion of terms justifies that it is convenient to analyze which are the most used and the subsequent analysis that we carried out. The most frequently found are those that include the different aspects from the EAS or EAI term: hippotherapy (103 occurrences), equine-assisted therapy (65), therapeutic horseback riding (16), and animal-assisted therapy (16). There are also related keywords to Physical Therapy (14) or Occupational Therapy (11). There is a variety of terms used to refer to this type of intervention and their evolution (EAS or EAI). A second group of descriptors refer to the target variables of the works, such as rehabilitation (20), balance (17) or postural balance (11), gross motor function (8), and mental health (7). Thirdly, those related to specific studied populations including Cerebral Palsy (47 cooccurrences), Autism Spectrum Disorder (18) or Autism (11), Multiple Sclerosis (13), and children (11) and child (6).

Bibliometric studies can be useful for both emerging and experienced researchers interested in the retrospective analysis of broad and diverse areas of research. Practical implications of the study include confirmation that EAS or EAI publications are of interest to researchers, publishers, and journals, as they are confirmed to be in a phase of exponential growth. In addition, countries/regions, collaborative networks, authors, journals, articles, authors’ keywords, and the most important research topics of interest have been identified. These research data favor collaboration between researchers, publishers, and journals, facilitating the location of experts and papers in the field, as well as journals potentially interested in manuscripts derived from this topic. 

This study has several limitations. The most important limitation was the possible selection bias since the data were only obtained from the WoS. Although this is the most widely used database for this type of study, future studies should include other databases such as PsycINFO and PubMed/Medline. Conducting less restricted searches, for example, using commands such as NEAR or focusing on some of the findings of this study, could also be other future research lines. Because of the increase in the research in this field, Refs. [44,45] with several systematic reviews, scoping reviews, etc. [7,36,40,46], this study may help future research and provide a starting point. Another limitation is that some authors may intentionally use uncontrolled words to increase the discoverability of their papers.

## 5. Conclusions

This bibliometric study shows interest in EAIs, as the exponential growth of publications, especially in recent years, has been confirmed. The WoS category where most papers were found was Rehabilitation, and the *Journal of Alternative and Complementary Medicine*, by Mary Ann Liebert, was the journal with the highest number of publications. The most cited paper was “State of the Evidence Traffic Lights 2019: Systematic Review of Interventions for Preventing and Treating Children with Cerebral Palsy”. The most prolific authors were Jeong-yi Kwon and Ji Lee, with the USA standing out among the 49 countries with the most co-authorships. The most used author keywords were rehabilitation, balance, and those related to specific populations such as CP and ASD. 

## Figures and Tables

**Table 1:** Web of Science categories with the greatest number of documents.

Web of Sciences Categories	Documents
Rehabilitation	84
Pediatrics	41
Clinical Neurology	36
Integrative Complementary Medicine	28
Psychiatry	24
Medicine General Internal	21
Sport Sciences	21
Veterinary Sciences	21
Neurosciences	20
Psychology Developmental	20
Public Environmental Occupational Health	17

**Table 2:** Prolific co-authors: networks, documents, and citations.

Authors	Network	N. Documents	N. Citations
Kwon, J.	Red	11	222
Lee, J.	Red	9	218
Wood, W.	Purple	9	113
Kim, Y.	Red	7	207
Peters, B. C.	Purple	6	65
Joung, Y.	Red	5	78
Shin, H.	Red	5	128
Silkwood-Sherer, D. J.	Yellow	5	187
Collado-Mateo, D.	-	4	54
Copetti, F.	-	4	86
Drache, M.	Sky blue	4	39
Fisher, P. W.	-	4	24
Gaviria, M.	Blue	4	17
Jang, B.	Red	4	72
Jormfeldt, H.	-	4	14
Krenek, N.	Green	4	97
Lanning, B. A.	Green	4	98
McGibbon, N. H.	Yellow	4	367
Poehlau, D.	Sky blue	4	39
Rigby, B. R.	Green	4	81
Shurtleff, T. L.	-	4	219
Song, J.	Red	4	72
Viruega, H.	Blue	4	17

“-”: without collaboration network between prolific co-authors. N.: number.

**Table 3:** Most cited papers.

Authors	Journal (ISO Abbreviation)	Cites	Year	WoS Index	WoS Categories
Novak et al.	*Curr. Neurol. Neurosci. Rep.*	356	2020	SCIE; SSCI	Clinical Neurology; Neurosciences
Bass et al.	*J. Autism Dev. Disord.*	218	2009	SSCI	Psychology, Developmental
Benda et al.	*J. Altern. Complement Med.*	145	2003	SCIE	Integrative and Complementary Medicine
Zadnikar et al.	*Dev. Med. Child Neurol.*	131	2011	SCIE	Clinical Neurology; Pediatrics
Johnson et al.	*Am. J. Health Promot.*	131	2009	SSCI	Public, Environmental and Occupational Health
Dewar et al.	*Dev. Med. Child Neurol.*	122	2015	SCIE	Clinical Neurology; Pediatrics
Gabriels et al.	*J. Am. Acad. Child Adolesc. Psychiatr.*	121	2015	SCIE; SSCI	Psychology, Developmental; Pediatrics; Psychiatry
Sterba et al.	*Dev. Med. Child Neurol.*	114	2007	SCIE	Clinical Neurology; Pediatrics
Shurtleff et al.	*Arch. Phys. Med. Rehabil.*	113	2009	SCIE	Rehabilitation; Sport Sciences
McGibbon et al.	*Dev. Med. Child Neurol.*	113	1998	SCIE	Clinical Neurology; Pediatrics
McGibbon et al.	*Arch. Phys. Med. Rehabil.*	101	2009	SCIE	Rehabilitation; Sport Sciences
Borgi et al.	*J. Autism Dev. Disord.*	88	2016	SSCI	Psychology, Developmental
Kwon et al.	*Arch. Phys. Med. Rehabil.*	80	2011	SCIE	Rehabilitation; Sport Sciences
Bronson et al.	*Eur. J. Phys. Rehabil. Med.*	77	2010	SCIE	Rehabilitation
Klontz et al.	*Soc. Anim.*	76	2007	SCIE; SSCI	Sociology; Veterinary Sciences
Tseng et al.	*Disabil. Rehabil.*	72	2013	SCIE; SSCI	Rehabilitation
Lechner et al.	*Arch. Phys. Med. Rehabil.*	71	2007	SCIE	Rehabilitation; Sport Sciences
Whalen et al.	*Phys. Occup. Ther. Pediatr.*	65	2012	SCIE; SSCI	Pediatrics; Rehabilitation
Kern et al.	*Altern. Ther. Health Med.*	65	2011	SCIE	Integrative and Complementary Medicine
Liptak et al.	*Ment. Retard. Dev. Disabil. Res. Rev.*	65	2005	SCIE; SSCI	Clinical Neurology; Neurosciences; Pediatrics; Psychiatry
Ajzenman et al.	*Am. J. Occup. Ther.*	64	2013	SSCI	Rehabilitation
Silkwood-Sherer et al.	*Phys. Therapy*	64	2012	SCIE	Orthopedics; Rehabilitation
Burgon et al.	*J. Soc. Work Pract.*	61	2011	SSCI	Social Work
Lanning et al.	*J. Autism Dev. Disord.*	59	2014	SSCI	Psychology, Developmental
Earles et al.	*J. Trauma Stress*	58	2015	SSCI	Psychology, Clinical; Psychiatry
Park et al.	*Yonsei Med. J.*	54	2014	SCIE	Medicine, General and Internal
Lechner et al.	*Spinal Cord*	52	2003	SCIE	Clinical Neurology; Rehabilitation
Kwon et al.	*J. Altern. Complement Med.*	50	2015	SCIE	Integrative and Complementary Medicine
Giagazoglou et al.	*Res. Dev. Disabil.*	50	2012	SSCI	Education, Special; Rehabilitation
Peters et al.	*J. Autism Dev. Disord.*	49	2017	SSCI	Psychology, Developmental
Stergiou et al.	*Am. J. Phys. Med. Rehabil.*	47	2017	SCIE; SSCI	Rehabilitation; Sport Sciences
Koca et al.	*North. Clin. Istanb.*	45	2015	ESCI	Medicine, General and Internal
Kendall et al.	*Eur. J. Psychother. Couns.*	43	2015	ESCI	Psychology, Clinical
Beinotti et al.	*Arq. Neuro-Psiquiatr.*	43	2010	SCIE	Neurosciences; Psychiatry
Rigby et al.	*J. Altern. Complement Med.*	38	2016	SCIE; SSCI	Integrative and Complementary Medicine
Trzmiel et al.	*Complement. Ther. Med.*	37	2019	SCIE; SSCI	Integrative and Complementary Medicine

WoS: Web of Science; Cites: times cited in the WoS Core; Year: publication year; SCIE: Science Citation Index Expanded; and SSCI: Social Science Citation Index.

**Table 4:** Bradford’s zones.

Zone	Number of Documents in Thirds (%)		Journals (%)		Bradford Multipliers	Journals (Theoretical Series)	
Core	99	30%	15	7%		15 × (n^0^)	15
Zone I	81	24%	35	17%	2.3	15 × (n^1^)	51
Zone II	153	46%	153	75%	4.4	15 × (n^2^)	173
Total	333	100%	203	100%	3.4		239
						% Error	−17.7%

**Table 5:** Core journals.

Publication Titles	Publisher	JCR Category 2022 (Quartile)	Doc.	Cit.
*Journal of Alternative and Complementary Medicine*	Mary Ann Liebert	Integrative and Complementary Medicine (Q3)	16	421
*Pediatric Physical Therapy*	Lippincott Williams & Wilkins	Pediatrics (Q3); Rehabilitation (Q3)	9	128
*Physiotherapy Theory and Practice*	Taylor & Francis	Rehabilitation (Q2)	8	67
*American Journal of Occupational Therapy*	Amer. Occupational Therapy Assoc.	Rehabilitation (Q1)	7	94
*Developmental Medicine and Child Neurology*	Wiley	Clinical Neurology (Q2); Pediatrics (Q1)	7	515
*International Journal of Environmental Research and Public Health*	MDPI	-	7	33
*Journal of Autism and Developmental Disorders*	Springer	Psychology, Developmental (Q1)	7	471
*Society & Animals*	Brill	Sociology (Q4); Veterinary Sciences (Q3)	6	114
*Archives of Physical Medicine and Rehabilitation*	Elsevier	Rehabilitation (Q1); Sport Sciences (Q1)	5	372
*Disability and Rehabilitation*	Taylor & Francis	Rehabilitation (Q2)	5	123
*Journal of Physical Therapy Science*	Soc. Physical Therapy Science	Rehabilitation (Q4)	5	79
*Physical & Occupational Therapy in Pediatrics*	Taylor & Francis	Pediatrics (Q3); Rehabilitation (Q2)	5	121
*Anthrozoos*	Taylor & Francis	Sociology (Q3); Veterinary Sciences (Q2)	4	78
*Complementary Therapies in Clinical Practice*	Elsevier	Integrative and Complementary Medicine (Q2)	4	60
*Multiple Sclerosis and Related Disorders*	Elsevier	Clinical Neurology (Q2)	4	18

JCR: journal citation report; Doc.: documents; Cit.: citations.

## Data Availability

Data is contained within the article or supplementary material.

## Supplementary Materials

The following supporting information can be downloaded at: https://www.mdpi.com/article/10.3390/ani14121776/s1.
